# A Consensus Compound/Bioactivity Dataset for Data-Driven Drug Design and Chemogenomics

**DOI:** 10.3390/molecules27082513

**Published:** 2022-04-13

**Authors:** Laura Isigkeit, Apirat Chaikuad, Daniel Merk

**Affiliations:** 1 Institute of Pharmaceutical Chemistry, Goethe University Frankfurt, 60438 Frankfurt, Germany; isigkeit@pharmchem.uni-frankfurt.de (L.I.); chaikuad@pharmchem.uni-frankfurt.de (A.C.); 2 Structural Genomics Consortium, BMLS, Goethe University Frankfurt, 60438 Frankfurt, Germany; 3 Department of Pharmacy, Ludwig Maximilian University of Munich, 81377 Munich, Germany

**Keywords:** big data, data curation, medicinal chemistry, machine learning, de novo design

## Abstract

Publicly available compound and bioactivity databases provide an essential basis for data-driven applications in life-science research and drug design. By analyzing several bioactivity repositories, we discovered differences in compound and target coverage advocating the combined use of data from multiple sources. Using data from ChEMBL, PubChem, IUPHAR/BPS, BindingDB, and Probes & Drugs, we assembled a consensus dataset focusing on small molecules with bioactivity on human macromolecular targets. This allowed an improved coverage of compound space and targets, and an automated comparison and curation of structural and bioactivity data to reveal potentially erroneous entries and increase confidence. The consensus dataset comprised of more than 1.1 million compounds with over 10.9 million bioactivity data points with annotations on assay type and bioactivity confidence, providing a useful ensemble for computational applications in drug design and chemogenomics.

## 1. Introduction

Information on small molecules and their biological activity extracted from scientific publications is currently available through several publicly accessible databases. Among these are large generic databases such as ChEMBL [1] and PubChem [2], which include “any” reported molecule and associated data, as well as specialized repositories focusing on extensively profiled “high-quality” compounds (BindingDB [3], IUPHAR/BPS [4], Probes & Drugs [5], Drugbank [6]). Such repositories are exceptionally valuable for medicinal chemistry, computer-aided drug design, pharmacology, and chemical biology. Since these databases have different objectives [7], their main focus, the contained data, the ways of data collection, and the level of details are diverse. The use of all available data is indispensable in some data-driven applications in life-science research and computer-aided drug design. Thus, a database that combines all publicly available information on compounds and their bioactivities would be valuable. For example, the assembly of a chemogenomics library containing the most active and selective compounds for hundreds of targets ought to rely on all available bioactivity information to capture potency and selectivity. Moreover, data-driven drug design techniques, e.g., machine learning, relying mainly on ChEMBL [8,9,10,11] or PubChem would profit from larger datasets that combine data from multiple repositories. Furthermore, data quality is another essential factor in decision-making regarding the use of public databases [12,13]. Combining information from multiple databases not only increases coverage of compound/bioactivity data, but may potentially reveal erroneous structural and activity data in a database [14,15] when comparing values from different sources, which thereby helps curating data in a semi-automatic fashion. Following these considerations, we have analyzed the overlap and gaps between several public databases of compound/bioactivity data and assembled a consensus database for chemogenomic compound search and machine learning applications. For rapid application, this database only contains information on chemical structure, biological target(s), and bioactivity/potency. Moreover, the database follows a common gene/target nomenclature and minimizes duplicate compound entries for different assays and targets. To add additional validation/confidence, it employs and compares structural and bioactivity information from various repositories.

## 2. Results

### 2.1. Sources of Compound/Bioactivity Databases

We used data from five sources for the assembly of the consensus database, including:

- ChEMBL28 [1] containing experimentally determined bioactivity data for 2.1 million drug-like bioactive molecules;
- PubChem [2] (downloaded 11.01.21) containing chemical and physical properties, as well as biological activities for more than 110 million molecules;
- BindingDB [3] (downloaded 25.02.21) containing experimentally determined binding affinities to biological targets for approx. 26,000 drug-like bioactive molecules;
- IUPHAR/BPS [4] Guide to Pharmacology (version 2021.1) containing curated information on biological targets and bioactivity for selected, pharmacologically active tool compounds;
- Probes & Drugs [5] (version 02b_2021) containing bioactivity data as well as target and signaling pathway information for more than 30,000 compounds from 29 public and commercial libraries, with great attention to chemical probes and drugs.

Except for PubChem, all described databases were fully incorporated into the custom database. Compounds from PubChem were only considered when they were included in at least one of the other databases. The data in PubChem for such compounds were used to validate and curate chemical structure and biological activities. Only compounds with a molecular weight of ≤1500 and an annotated biological activity on a human target were included, resulting in the following numbers of molecules per source database:

- ChEMBL28: 1,131,947 molecules;
- PubChem: 444,152 molecules;
- BindingDB: 26,856 molecules;
- IUPHAR/BPS: 7371 molecules;
- Probes & Drugs: 34,211 molecules.

### 2.2. Analysis of Public Compound/Bioactivity Databases

Before assembling a consensus database from the publicly available compound/bioactivity data, we evaluated the selected source databases. We extracted and compared the contained molecules to reveal unique and shared compound entries, and we analyzed the distribution of bioactivities as well as the coverage of large protein target families (e.g., kinases, G protein-coupled receptors, nuclear receptors, transporters, ligand-gated ion channels, cytochrome P450, and writers/readers/erasers). In addition, we used molecular descriptors (e.g., molecular weight, number of rotatable bonds, number of aromatic bonds, and cLogP) and analyzed the molecular scaffold diversity to compare the source databases.

#### 2.2.1. Database Coverage and Overlap

**Figure 1:**
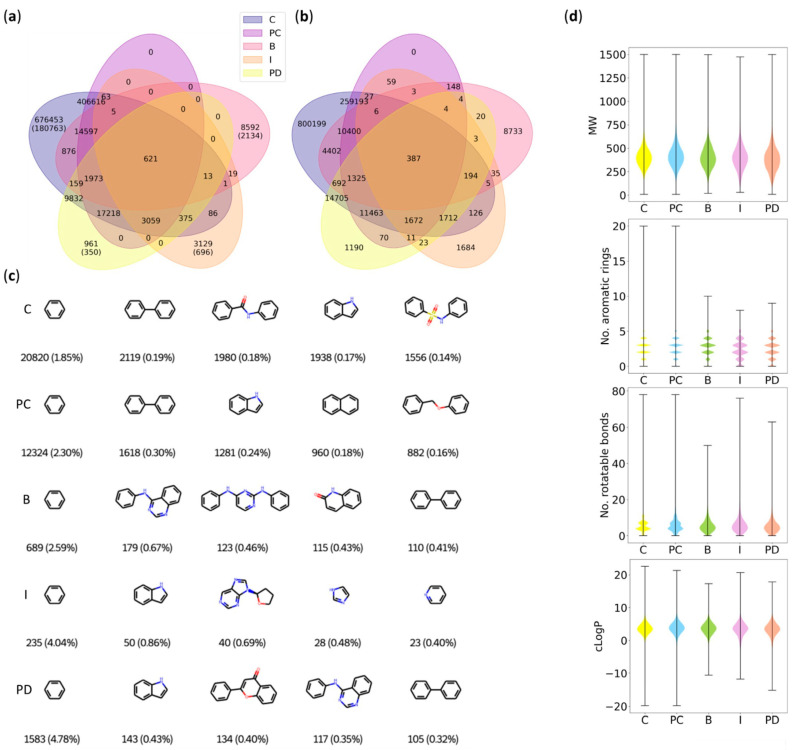

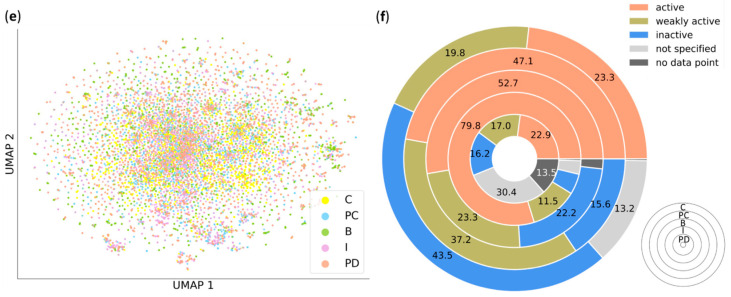
Analysis of individual compound/bioactivity databases to assemble a consensus dataset (C = ChEMBL, PC = PubChem, B = BindingDB, I = IUPHAR/BPS, and PD = Probes & Drugs). From PubChem, only compounds that were also included in at least one of the other databases were considered and the data in PubChem referring to these compounds were used to validate and curate chemical structure and biological activities. (**a**,**b**) Venn diagrams of the data collected from the five different databases and created by compound ID (**a**) or SMILES (**b**). The numbers of shared and not-shared molecules are reported and the numbers of unique scaffolds are in round brackets. (**c**) The five-most frequently occurring Murcko scaffolds present in the databases. (**d**) Distribution of molecular weight (MW), number of aromatic rings, rotatable bonds, and octanol–water partition coefficients (cLogP) per database. (**e**) UMAP of 2000 randomly selected molecules from each database. (**f**) Percentage pie chart of records labeled as active (activity log-value higher than 6), weakly active (activity log-value between 5 and 6), inactive (activity log-value lower than 5 or labeled inactive), not specified (no activity log-value), and no data point. Each ring represents a database in the order shown in the legend. Percentages below 5% are not displayed.

To reveal the overlapping information (common compounds) and potential differences/gaps in the coverage of the public databases, we compared the compound IDs (PubChem CIDs and ChEMBL IDs, see also Section 2.3) as well as the molecular structures using canonical Simplified Molecular Input Line Entry System (SMILES) [16] strings for all databases (Figure 1a). Surprisingly, only 39.8% (455,513 of 1,144,648) of the molecules were contained in more than one source database and only 0.14% (621) of these molecules were found in all sources. Only 64.9% (295,404) of the molecules contained in at least two databases had the same canonical SMILES deposited in the different repositories. Database comparison purely based on canonical SMILES revealed only 306,689 compounds shared by two or more databases and only 387 compounds shared by all (Figure 1b). Combining the information content from all the public databases, hence appeared attractive.

#### 2.2.2. Compound Space of Databases

Murcko scaffold analysis [17] revealed 353,492 unique scaffolds in total, among which, 169,883 (48.1%) were shared by two or more databases. Only 449 (0.3%) scaffolds were present in all databases. Conversely, each source thus contributed unique atomic scaffolds. For example, 2134 of the 8592 molecules (24.8%) contained only in the BindingDB represented novel scaffolds (ChEMBL: 180,763 of 676,453 (26.7%); IUPHAR: 696 of 3129 (22.3%); Probes & Drugs: 350 of 961 (36.4%)). The IUPHAR/BPS and Probes & Drugs databases had the highest diversity as observed by high percentages of different scaffolds with 58.7% (4327 different scaffolds in 7371 contained molecules) and 52.5% (17,963 different scaffolds in 34,211 contained molecules), respectively. For the remaining databases, a lower diversity was observed with 31–36% unique scaffolds per compound ratio. This observation further supports the need for a consensus database and highlights unique contributions from all data sources. Benzene emerged as the most common scaffold in all databases, and biphenyl and indole were also frequent (top 5) (Figure 1c).

Despite large differences in terms of the contained compounds and scaffolds, the distributions of common drug-like features such as molecular weight, number of aromatic bonds, number of rotatable bonds, and predicted octanol–water partition coefficients (Figure 1d) were similar across the sources. Owing to the largest number of contained molecules, the ChEMBL database had the widest distribution and the highest frequency of outliers compared to the other databases, while the more focused databases IUPHAR/BPS, BindingDB, and Probes & Drugs showed narrower distributions. Interestingly, molecules annotated in IUPHAR/BPS tended to comprise fewer aromatic rings. Molecular similarity analysis by extended connectivity fingerprints (ECFP, 1024 bits, radius = 2) [18] and uniform manifold approximation and projection for dimension reduction (UMAP) [19] also demonstrated a similar chemical space coverage of the source databases, despite their incomplete overlap (Figure 1e).

#### 2.2.3. Activity Analysis

**Figure 2:**
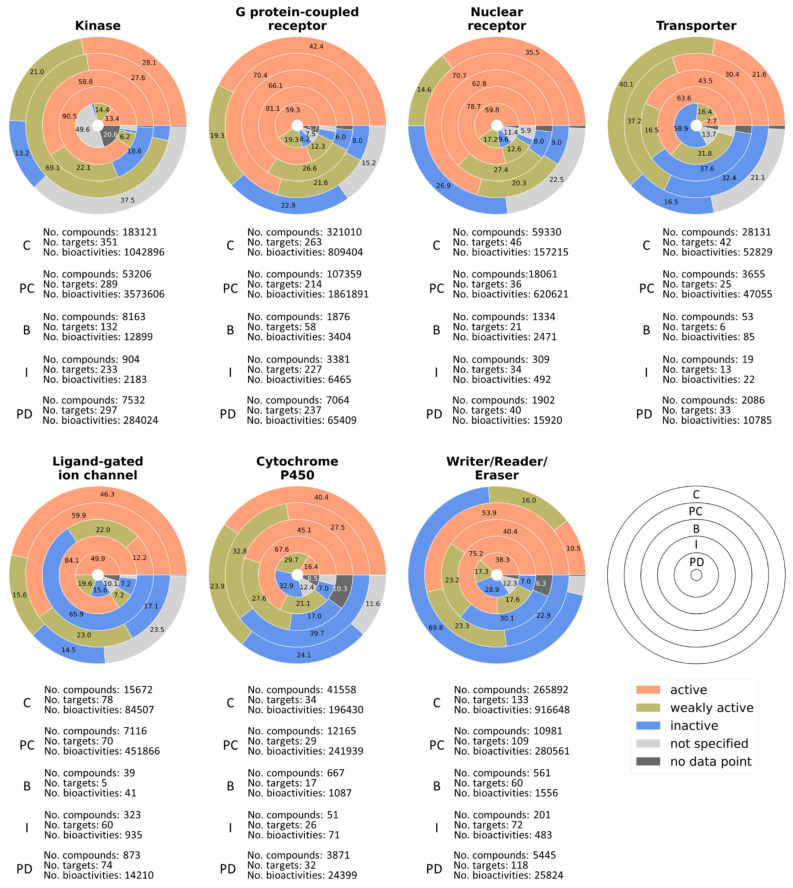
Percentage pie chart of the bioactivity labels for seven important target families and detailed numbers of associated targets, compounds, and bioactivities (C = ChEMBL, PC = PubChem, B = BindingDB, I = IUPHAR/BPS, and PD = Probes & Drugs). Each ring represents a database in the order shown in the legend. Percentages below 5% are not displayed.

We also evaluated and compared the activity landscape/distribution of the compounds from the different databases. For this, all annotated bioactivity data were classified by their potency and assigned one of the bioactivity labels of either “active”, “weakly active”, “inactive”, “no data point”, or “not specified”, which were inspired by previous work [20]. Experimental bioactivities with a log-value greater than six or with an assay comment “active” were classified as “active”. Bioactivity annotations with a log-value between five and six were assigned to the “weakly active” group. The “inactive” label denoted bioactivities with a log-value below five or with the assay comment “inactive”. Where no value was present, the label “no data point” was added, and all other bioactivities were labeled as “not specified”. Comparison of the source databases in terms of activity distribution (Figure 1f) and coverage of large target families (Figure 2) demonstrated that each source contributed differently to the final consensus dataset.

- In ChEMBL, there were 6,575,449 annotated bioactivities covering 4081 targets for the 1,131,947 selected molecules, of which 23.3% were active, 19.8% weakly active, 43.5% inactive, 13.2% not specified, and 0.2% had no data point;
- In PubChem (for comparison/curation), there were 11,587,761 annotated bioactivities on 3199 targets for 444,152 selected molecules, of which 47.1% were active, 37.2% weakly active, 15.6% inactive, 0.01% not specified, and 0% had no data point;
- In BindingDB, there were 51,424 annotated bioactivities on 758 targets for 26,856 molecules, of which 52.7% were active, 23.3% weakly active, 22.2% inactive, 0% not specified, and 1.8% had no data point;
- In IUPHAR/BPS, there were 15,557 annotated bioactivities covering 1657 targets for 7371 molecules, of which 79.8% were active, 11.5% weakly active, 5% inactive, 3.3% not specified, and 0.5% had no data point;
- In Probes & Drugs, there were 930,209 bioactivities on 4042 targets for 34,211 molecules, of which 22.9% were active, 17% weakly active, 16.2% inactive, 30.4% not specified, and 13.5% had no data point.

This analysis aligned with the focus of the different source databases. ChEMBL provided the most bioactivity tags, including the most inactive labels. However, there were also many unspecified, non-traceable bioactivity marks. IUPHAR/BPS focusing on potent, expert-selected chemical tools contained the highest frequency of active labels. Probes & Drugs additionally emphasizes on selectivity information and hence had the highest bioactivity information per compound ratio, many of which were not specified, however. PubChem showed a high ratio of specified bioactivity data, which was very useful for curation.

Information on biological targets and target families is another critical aspect for a bioactivity database. ChEMBL provides valuable information on target families, which was extracted and applied to the other databases in order to assign each target a family. Analysis of the coverage of seven large pharmacologically relevant target families in terms of number of compounds, targets, bioactivities, and distribution of bioactivity labels revealed further key differences in the source databases (Figure 2). ChEMBL had the greatest coverage of all seven target families in terms of covered targets and active compounds but contained a high proportion of unspecified activities. The greatest number of bioactivities was obtained from PubChem, although it was only used to add information to molecules contained in at least one of the other sources. Most bioactivities were retrieved for kinases followed by G protein-coupled receptors, potentially due to the large size of these protein families. Overall, the different coverage provided by diverse source databases further supports the benefit of a consensus dataset.

### 2.3. Consensus Database Assembly

The analysis of public compound/bioactivity databases (Section 2.2) demonstrated remarkable differences in terms of the numbers of molecules, annotated bioactivities, and target coverage, supporting a need for a consensus database. To compile such a set, we gathered the entire public databases, which were provided in different formats, and fed them into the Konstanz Information Miner (KNIME) [21]. The datasets from different sources were then prepared for merging by unifying the data format, standardizing target names according to the HUGO Gene Nomenclature Committee (HGNC) [22], flagging assay types (cell-based, cell-free, functional, and unspecified) by keywords, and converting molar activity/affinity values to a negative decadic logarithm format where possible. Only molecules with a molecular weight below 1500 and bioactivity on a target of the organism homo sapiens were considered. After this pre-processing, the records of each database were grouped based on their compound IDs (ChEMBL ID or PubChem CID). Structural information (e.g., SMILES) was not used for merging to enable a structure check for the grouped compound entries. After grouping by compound IDs, information on targets, activity types, and assay types was grouped to provide an overview of the activity values for each compound–target pair. Activities for the same compound on different targets were retrieved for 2.9% of the contained compounds and were kept separate. This small fraction of compounds occurring in more than one line (as different compound–target pairs) indicated that the number of true duplicates—if any—was very small. Bioactivities referring to the same compound–target pair were grouped and compared. For multiple annotated bioactivities for a compound–target pair within one log unit difference (e.g., <10-fold), their mean was calculated and kept together with the number of the underlying values. When the difference was more than one log unit, each annotated bioactivity was kept separately in multiple columns. The databases were then merged into a consensus dataset by aggregating compound/bioactivity data based on compound IDs (ChEMBL ID, PubChem CID), gene symbols, activity type, assay type and unit. After this, a bioactivity check was performed by comparing values from the different sources and adding an activity check annotation to provide automated activity validation for additional confidence. In addition, we performed a molecular structure check and compared the structural information stored in the databases after harmonization of structural information from different sources [23]. For this task, we removed salts from the molecular structures, created canonical SMILES, and canonicalized tautomers for each entry from all databases, and checked if the SMILES matched. We introduced a “Structure check (Tanimoto)” column to denote matching or differing compound structures and to give information about the similarity between non-matching structures.

**Figure 3:**
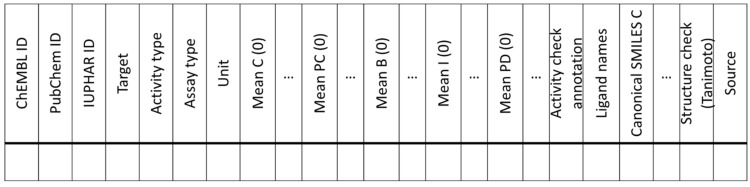
Structure of the consensus dataset.

Previous work to assemble a target-focused dataset for nuclear receptor ligands (NURA) [20] inspired the structure of this dataset. The consensus dataset comprises the following information and the format exemplified in Figure 3.

- Compound information: ChEMBL ID, PubChem ID, IUPHAR/BPS ID, and all unique names contained in the five source databases are listed. The molecular structure is expressed as canonical SMILES. The biological target is given using HGNC symbols and the corresponding target family;
- Experimentally determined bioactivity (including unit of measure, activity type, assay type), pivoted/grouped by the ligand, target, unit of measure, activity type, and assay type. For IUPHAR/BPS, BindingDB, and Probes & Drugs, the assay type is defined by the activity type (K_i_, K_d_ = cell-free; IC_50_, EC_50_ = cell-based) and for ChEMBL and PubChem through keyword search in the assay description (cell-free: e.g., “binding affinity”, “recruitment”, “displacement”, “fret”, “htrf”; cell-based: e.g., “cell” in combination with “reporter” or “receptor”, “transfection”, “transactivation”, “luciferase”, “galactosidase”; functional: e.g., “cell proliferation”, “cell” without “reporter” or “receptor”; unspecified: any other). After pivoting, the dataset has multiple columns representing different bioactivities from the different databases and up to four rows for different assay types per compound/target pair. In addition, the number of activity values for the respective compound/target pair in the respective database is noted;
- Activity check annotation: For automated curation and to add confidence on annotated bioactivity data, bioactivity values with common activity type and assay type from different source databases were compared and flagged as: no comment: bioactivity values are within one log unit; check activity data: bioactivity values are not within one log unit; only one data point: only one value was available, no comparison and no range calculated; no activity value: no precise numeric activity value was available; no log-value could be calculated: no negative decadic logarithm could be calculated, e.g., because the reported unit was not a compound concentration;
- Structure check (Tanimoto): To denote matching or differing compound structures in different source databases, we added a structure check label as follows: match: molecule structures are the same between different sources; no match: the structures differ; one structure: no structure comparison was possible, because there was only one structure available; no structure: no structure comparison was possible, because there was no structure available. For structure annotations that did not match, we computed the Jaccard–Tanimoto similarity coefficient on Morgan fingerprints [24] for sets of structures for one compound from the different sources to reveal true structural differences. The minimum similarity value is presented in the final dataset together with the structure check.

### 2.4. Analysis of the Consensus Database

**Figure 4:**
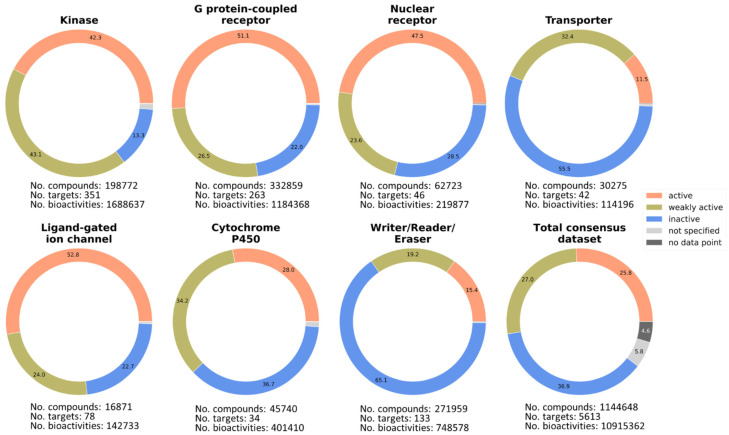
Percentage pie chart of the bioactivity labels of the complete consensus dataset for seven important target families with detailed numbers of associated targets, compounds, and bioactivities.

The final dataset contains 1,144,648 molecules with 10,915,362 bioactivities on 5613 targets (including isoforms, cell lines, phenotypic experiments, etc.) with an activity distribution of 25.8% active, 27% weakly active, 36.9% inactive, 5.8% not specified, and 4.6% with no data point (Figure 4). A total of 5,779,130 (52.9%) bioactivities were retrieved from cell-free, 4,031,770 (36.9%) from cellular, and 917,120 (8.4%) from functional assays. A total of 187,342 (1.7%) referred to other or unspecified test systems. A total of 51,912 (0.5%) bioactivity annotations differed in different sources according to our threshold of one log unit and were flagged. Figure 4 further shows that the number of compounds and bioactivity data for the seven important target families increased in the consensus dataset.

Detailed analysis of the activity check annotation for bioactivity data retrieved from more than one source (1,352,614 bioactivities; Table 1) showed an exact match for 987,022 (73%) bioactivity annotations, while 45,831 (3.4%) values were within the range of one log unit. A total of 51,912 (3.8%) reported bioactivities were outside this range and hence were considered as not matching. A total of 192,951 (14.3%) bioactivity annotations only referred to a comment with no precise value, and no log value could be calculated for 74,898 (5.5%). The bioactivity check could thus not be performed for these data.

**Figure 5:**
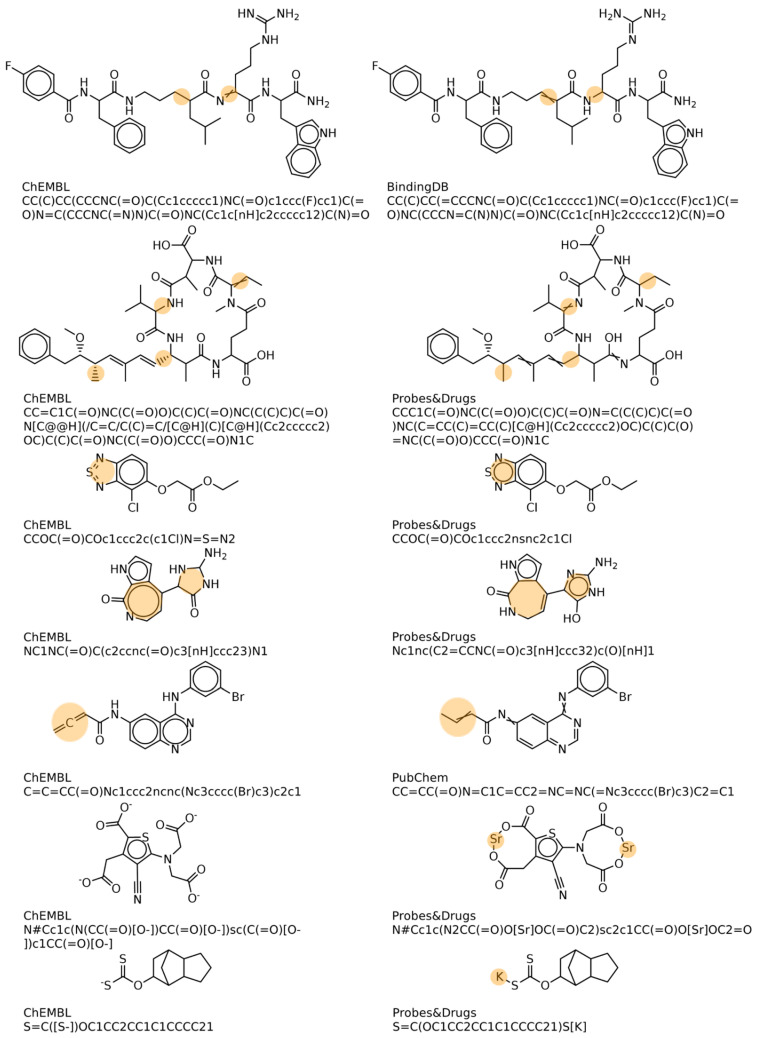
Differences in structural data from different source databases (examples).

A total of 9,562,748 (87.6%) bioactivities were only available from one source and no SMILES string was documented for 110,947 (0.9%) bioactivities; thus, no structure check could be performed. Among the bioactivities annotated in at least two source databases (1,352,614), 1,322,851 (97.8%) were for the same canonical SMILES and 29,763 (2.2%) referred to different canonical SMILES. A total of 65% (19,510) of entries with differences in the SMILES string revealed a Jaccard–Tanimoto similarity computed on Morgan fingerprints of one, indicating no relevant structural difference. In the remaining 10,253 entries with different SMILES and a similarity <1, structural differences were due to different or missing stereochemical information, tautomers that were not normalized in the preprocessing (mostly involving sulfur atoms as center), salt and complex bonds that were annotated as covalent bonds in the source data, and true structural differences/errors (examples are shown in Figure 5).

## 3. Discussion

The diverse publicly available compound/bioactivity databases constitute a key resource for data-driven applications in chemogenomics and drug design. Analysis of their coverage of compound entries and biological targets revealed considerable differences, however, suggesting a benefit of a consensus dataset. Therefore, we combined and curated information from five esteemed databases to assemble a consensus compound/bioactivity dataset comprising 1,144,648 compounds with 10,915,362 bioactivities on 5613 targets (including defined macromolecular targets as well as cell-lines and phenotypic readouts). The dataset provides simplified information on assay types underlying the bioactivity data as well as a bioactivity confidence notice and a structure check based on the comparison of data from different sources. Interestingly, this automated curation and evaluation procedure revealed a considerable number of relevant differences in the structural data provided by different sources.

We unified the source databases, brought them into a common format, and combined them, easing its use for multiple generic applications such as chemogenomics and data-driven drug design. Nevertheless, there remain some limitations. Importantly, a considerable fraction of bioactivity data in the consensus dataset were only found in one source database, mainly ChEMBL, obscuring automated comparison and curation in these cases. In addition, ChEMBL contains many units of measurement and activity types that are not found in the other databases, thus preventing further automated comparison. Our keyword-based classification of assay types additionally may not be optimal for the PubChem and ChEMBL databases, therefore, some matching bioactivities were not merged. Conversely, the automated approach towards assay classification and matching of bioactivities may have merged data from incomparable assay setups in some cases. The flags in the bioactivity check should therefore be treated with some care and manually examined in cases where precise bioactivity data matter. Another limitation may arise from using compound IDs for matching. Although the number of duplicates after merging was small and the structural information matched for a very large fraction of molecules in the consensus set, which was merged based on the compound IDs, the IDs might have not been fully up-to-date and consistent in all source databases, which may have led to incomplete merging.

Still, our results show that the consensus dataset provides increased target coverage and contains a higher number of molecules compared to the source databases, which is also evident from a larger number of scaffolds. These features render the consensus dataset a valuable tool for machine learning and other data-driven applications in (de novo) drug design and bioactivity prediction. The increased chemical and bioactivity coverage of the consensus dataset may improve robustness of such models compared to the single source databases. In addition, semi-automated structure and bioactivity annotation checks with flags for divergent data from different sources may help data selection and further accurate curation. The final consensus dataset is available at 10.5281/zenodo.6398019.

## 4. Materials and Methods

### 4.1. Data Collection and Database Merging

Data from all source databases were curated by a common procedure with the following sequential steps:

1. Download the databases and the additional compound identifier mapping information between the different databases (ChEMBL ID, PubChem CID, and UniProt ID) and loading all data into the Konstanz Information Miner (KNIME);
  - ChEMBL downloaded as PostgreSQL database;
  - PubChem downloaded as RDF-files;
  - BindingDB downloaded as SDF-file;
  - IUPHAR/BPS downloaded as CSV-file;
  - Probes & Drugs downloaded as PostgreSQL database.
2. Mapping the compounds to the compound identifier information;
3. Only records for the organism homo sapiens and for compounds with a molecular weight lower than 1500 were considered;
4. Standardization of target names using HGNC (gene symbols);
5. Calculation of the negative decadic logarithm of experimental readouts with a molar unit; all other experimental readouts with other units were not changed;
6. Classification of records from ChEMBL and PubChem into cell-free, cell-based, and functional using keywords from the assay description (assay type); for IUPHAR/BPS, BindingDB, and Probes & Drugs, the activity type defined the assay type;
7. Pivoting the data of each source database first by compound ID (ChEMBL ID, PubChem ID) and then pivoting data referring to one molecule by gene symbol, activity type, assay type, and unit. After that, the ranges of log-values were calculated:
  - range ≤ 1 -> calculate mean of log-values + frequency;
  - range > 1 -> split into several columns + frequency;
  - no activity value deposited -> searching for information in assay comments and write them in columns + frequency.
8. Merging the different databases based on their compound ID (ChEMBL ID, PubChem ID), gene symbol, activity type, assay type, and unit;
9. Activity check annotation between databases: Calculating range between highest and lowest value in the new dataset:
  - range ≤ 1 -> no comment;
  - range > 1 -> ‘check activity data’;
  - range not calculable -> ‘only 1 data point’;
  - no activity value deposited -> ‘no activity value’;
  - no log-value -> ‘no log-value could be calculated. Please check the matches by yourself’.
10. Salts were removed from the molecular structures and the remaining structures were converted into canonical SMILES strings. Lastly, the molecules were screened for tautomers and the canonical tautomer was generated.
11. Structure check: The structure check was performed on the standardized SMILES by string comparison. Only for entries that did not match, we compared the structures with the Jaccard–Tanimoto coefficient computed on Morgan fingerprints and noted the minimum value.

### 4.2. Software and Code

Data aggregation and curation were performed in KNIME 4.3.3 [21]. Salts were removed and SMILES were canonicalized using the nodes ‘RDKit Salt Stripper’ and ‘RDKit Canon SMILES’. Extended connectivity fingerprints (ECFPs) were calculated in Python v3.7 with RDKit 2021.03.4 with the following settings: “Bits per pattern” = 1024 and “radius” = 2. Morgan Fingerprints and the Jaccard-Tanimoto similarity coefficient were calculated in Python v3.7 with RDKit 2021.03.4. Canonical tautomer generation was done with Python v3.7 using RDKit 2021.03.4. UMAP projection was done with Python v3.7 and the module umap-learn v0.5.2 with the following parameters: “n_neighbors” = 500, “min_dist” = 0.99, “metric” = correlation, “random_state” = 16. Murcko scaffolds, molecular weight, number of aromatic rings, rotatable bonds, and octanol–water partition coefficients were computed with RDKit 2021.03.4 in Python v3.7 with default settings. The Venn diagrams were created using the module venn v0.1.3. in Python v3.7.

## Figures and Tables

**Table 1:** Analysis of activity check annotation for bioactivities in at least two source databases. Percentage values refer to bioactivities that were found in at least two sources.

Category	Total Number	Percentage
Exact match of the activity values	987,022	73
Match within one log change	45,831	3.4
Outside one log change (no match)	51,912	3.8
No activity value	192,951	14.3
No log value could be calculated	74,898	5.5

## Data Availability

The consensus dataset generated and analyzed in this study is freely available from Zenodo (https://zenodo.org), with the doi:10.5281/zenodo.6398019.

## Abbreviations

HGNC	HUGO Gene Nomenclature Committee at the European Bioinformatics Institute
KNIME	Konstanz Information Miner; SMILES, Simplified Molecular Input Line Entry System
SMILES	Simplified Molecular Input Line Entry System
UMAP	uniform manifold approximation and projection for dimension reduction
